# Waldenström macroglobulinemia presenting as bilateral bloody pleural effusion: A case report

**DOI:** 10.1097/MD.0000000000038406

**Published:** 2024-06-14

**Authors:** Tiantian Cen, Qiaoli Zhang, Yanan Ying, Zhongbo Chen, Xiaoqun Zhang, Xiao Wu, Qian Deng, Gun Chen, Fengyun Tao, Peipei Ye, Hongying Ma

**Affiliations:** a Department of Respiratory and Critical Care Medicine, Key Laboratory of Respiratory Disease of Ningbo, The First Affiliated Hospital of Ningbo University, Ningbo, Zhejiang, China; b Department of Nephrology, Key Laboratory of Respiratory Disease of Ningbo, The First Affiliated Hospital of Ningbo University, Ningbo, Zhejiang, China; c Department of Hematology, Key Laboratory of Respiratory Disease of Ningbo, The First Affiliated Hospital of Ningbo University, Ningbo, Zhejiang, China; d Department of Tissue Pathology, Ningbo Clinical Pathology Diagnosis Center, Ningbo, China; e Department of Pathology, Ningbo Yinzhou People’s Hospital, Ningbo, China; f Department of Hematology, Ningbo Yinzhou People’s Hospital, Ningbo, China.

**Keywords:** case report, pleural effusion, Waldenström macroglobulinemia

## Abstract

**Rationale:**

Pleural effusion, especially bilateral bloody pleural effusion, is a rare complication of Waldenström macroglobulinemia (WM). Pleural effusion in patients with WM has many causes, such as infection, tumor invasion of the pleura, and rupture of the thoracic duct or its branches. Patients with WM presenting to the respiratory department with chest tightness and shortness of breath need more differential diagnosis by respiratory physicians, which is helpful for effective treatment. Herein, we present a case of MV diagnosis in a patient with bilateral bloody pleural effusion.

**Patient concern:**

Our patient is a 59-year-old man with WM presenting as having bilateral bloody pleural effusion.

**Interventions:**

The patient was treated with pleural effusion drainage. After confirming the diagnosis, the patient was treated with rituximab, cyclophosphamide, and dexamethasone.

**Outcomes:**

Following these treatments, the patient’s symptoms improved, and ultrasound showed a decrease in pleural effusion.

**Lessons:**

Despite its favorable prognosis, the cause of pleural effusion in a patient with WM can be challenging to diagnose. The cause of pleural effusion should be considered a differential diagnosis when diagnosing patients diagnosed with WM.

## 1. Introduction

Waldenström macroglobulinemia (WM) manifests physically as lymphadenopathy (15%), splenomegaly (15%), and hepatomegaly (20%).^[1]^ The most typical symptom associated with normocytic anemia is weariness.^[2]^ Pulmonary and pleural involvement due to the disorder is relatively rare, only occurring in 3% to 5% of all extramedullary cases.^[3]^ One extremely uncommon sign of WM is bloody pleural effusion, mainly when it occurs bilaterally.^[4]^ This report details a case of bilateral bloody pleural effusion that was the symptom of WM.

## 2. Case report

**Figure 1.**
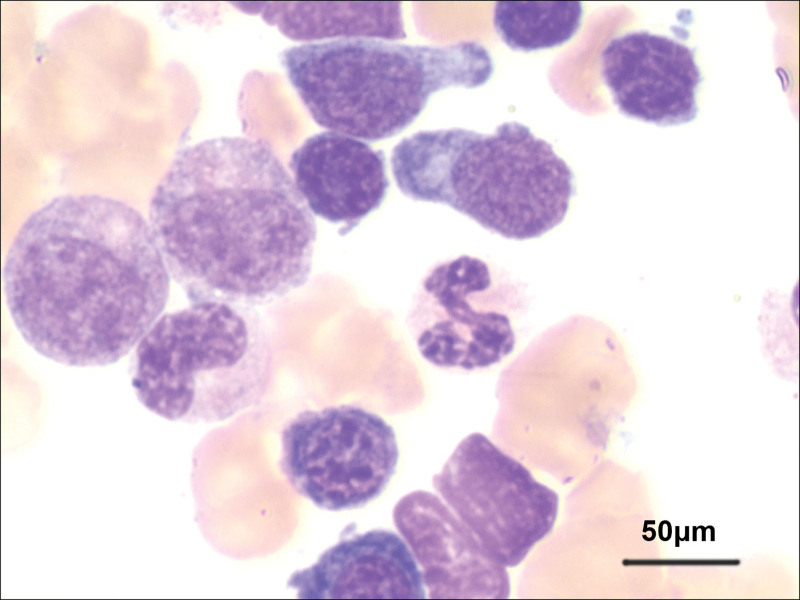
Microscopic view of bone puncture pathology, 400×.

A 59-year-old man was admitted to our hospital due to chest tightness and dyspnea after a cold, which was aggravated after a quick walk or climbing stairs. He had a fever 1 week ago, with a maximum temperature of 39 °C. In Addition, he had mild pitting edema in both lower limbs. Nine months ago, the patient was diagnosed with WM by biopsy pathology of bone marrow aspiration in another hospital (Fig. 1) and was treated with zanubrutinib until now. Monoclonal IgM was detected in the serum. Plasmacytoid lymphocytes in the bone marrow showed trabecular space invasion. Abnormal CD19 (+), CD5 (−), and CD10 (−) mature monoclonal B lymphocytes (3.87%) and abnormal monoclonal plasma cells (1.44%) were found in the bone marrow. He had no other medical, family, or psychosocial history including comorbidities, or relevant genetic information.

**Figure 2.**
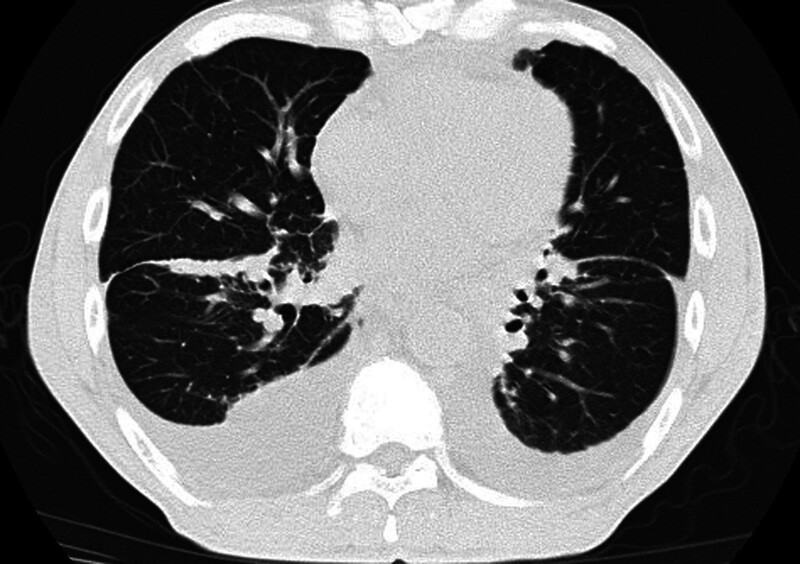
Chest CT scan-axial view.

**Figure 3.**
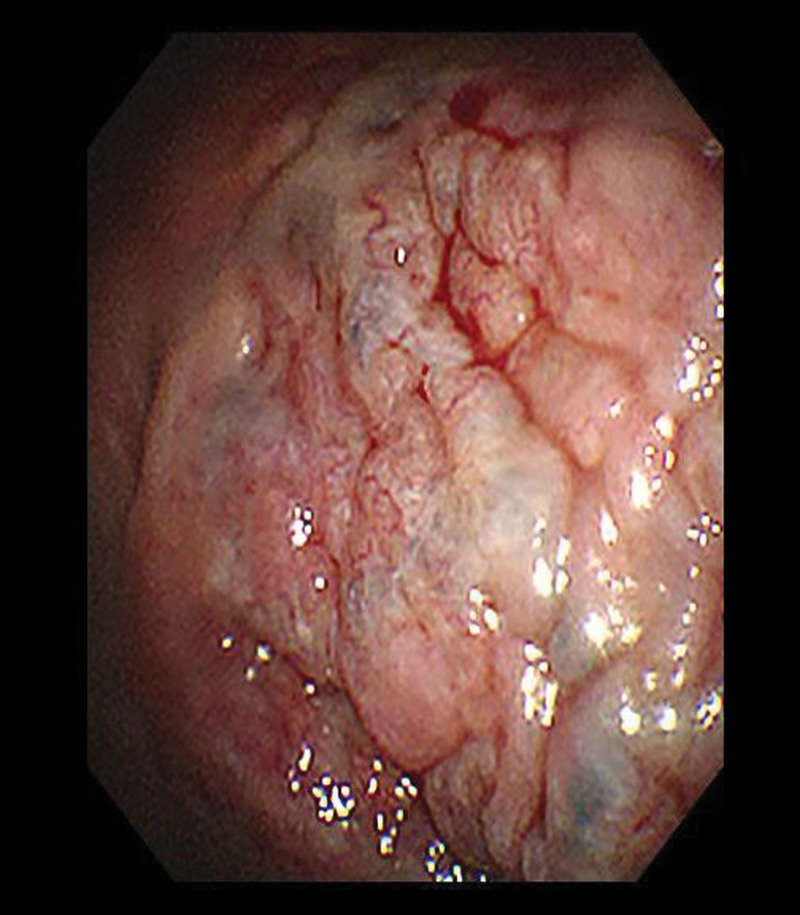
Visceral pleura.

**Figure 4.**
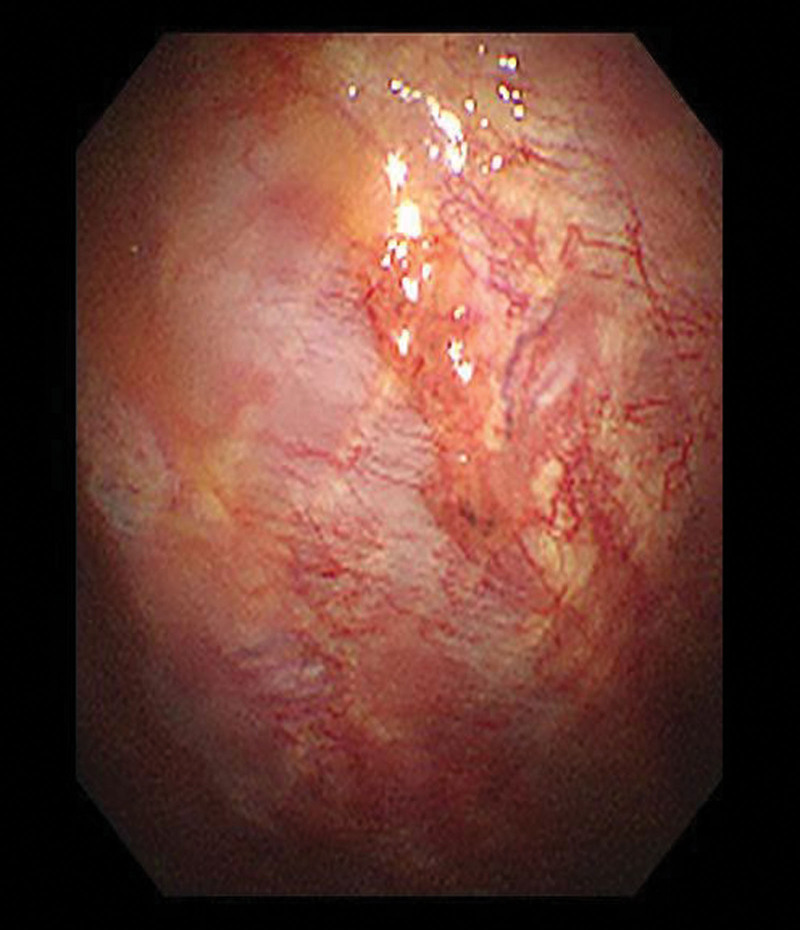
Parietal pleura.

**Figure 5.**
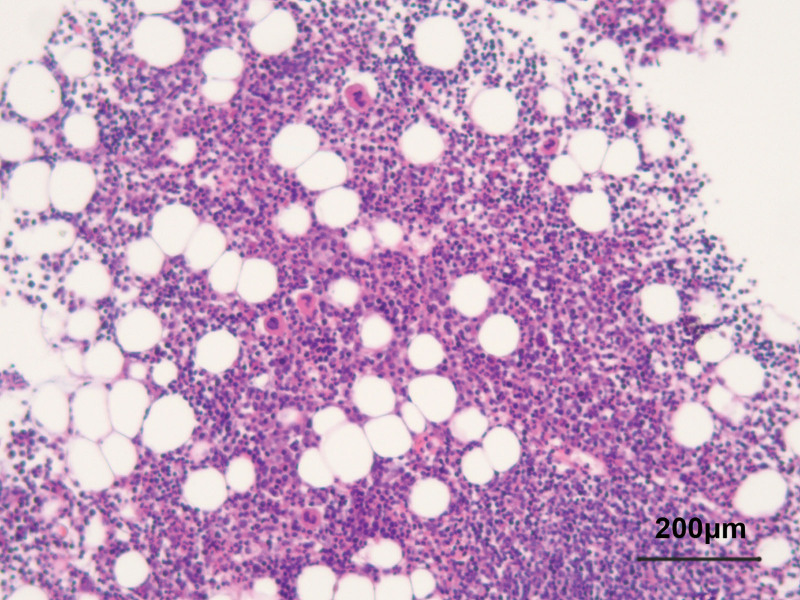
Microscopic view of pleural biopsy pathology, 100×.

On admission, a physical examination indicated reduced breath sounds, primarily in the right lung fields, and mild pitting edema in both lower limbs. The blood analysis revealed signs of anemia (Hb 10.6 mg/dL), C-reactive protein, and WBC in the normal range. The blood sedimentation rate was 120 mm/h. Inflammatory markers showed Interleukin-6 10.06 pg/mL. Immune globulin in blood showed IgM 31.80 g/L, LAM light chain 26.20 g/L, IgG 5.84 g/L, IgA 0.89 g/L, complement 3 0.58 g/L, KAP light chain 4.35 g/L (normal levels: 0.3–2.2, 3.13–7.23, 8.60–17.40, 1.00–4.20, 0.70–1.40, 6.29–13.50 g/L, respectively). In addition, IgE and complement 4 are in the normal range. The chest CT showed bilateral pleural effusion and exudation (Fig. 2). The chest ultrasound showed bilateral pleural effusion occupying about 3 intercostal spaces. We performed bilateral therapeutic thoracentesis with drainage of 1050 mL of bloody cloudy fluid. Pleural fluid resulted as a transudate with erythrocytes 113,000/µL, a positive Rivalta test (protein 33.1 g/L, pleural fluid/serum protein ratio 0.51, pleural fluid/serum LDH ratio 1.00, serum LDH 108 U/L, fluid LDH 108 U/L). The pleural effusion was exudative and predominantly composed of lymphocytes (87%). Microbiological tests were negative. We also sent a pleural fluid sample to the hematology laboratory to perform an immunophenotyping with monoclonal antibodies. Flow cytometry showed about 11.76% monoclonal B cells, called abnormal B-cell clones. Then, a thoracoscopic pleural biopsy was performed. Thoracoscopy showed nodular changes in the right visceral pleura and local yellowish ridges on the parietal pleura (Figs. 3 and 4). Pleural biopsy was performed, and amyloidosis was considered (Fig. 5). Immunohistochemistry of pleural biopsy showed Congo red (+), hexamine silver (−), Masson (+), CD 138 (+), and CD38 (+). Then, the patient was given zebrutinib 160 mg/day orally to control WM. After discharge, the patient was treated with rituximab, cyclophosphamide, and dexamethasone. Then, the symptoms of the patient improved, and the ultrasound showed a decrease in pleural effusion.

## 3. Discussion

WM is a distinct B-cell lymphoproliferative illness characterized by lymphoplasmacytic lymphoma (LPL) invasion of the bone marrow and immunoglobulin IgM monoclonal gammopathy.^[5]^ Pleural effusion is the most common symptom of respiratory diseases, but WM with bilateral bloody pleural effusion is rare. There are many causes of pleural effusion in patients with MV, and different types of pleural effusion patients have different treatment priorities.

It has been reported that ibrutinib and other tyrosine kinase inhibitors cause pericardial and pleural effusions.^[6]^ Peripheral edema was the most often reported side effect (14%) in a meta-analysis of 6 phase III clinical trials with ibrutinib and other tyrosine kinase inhibitors.^[7]^ It is unclear how ibrutinib or other tyrosine kinase inhibitors, like dasatinib, cause peripheral edema and effusions in patients with chronic myelogenous leukemia. However, it is thought to be related to immune cells’ disrupted downstream signaling, which may allow regulatory T cells to be suppressed, causing inflammation and microvascular permeability.^[8]^ Although pleural effusions caused by tyrosine kinase inhibitors are also lymphocyte-dominated exudates, there were no malignant findings on pathology.^[9]^ In addition, pleural effusions caused by tyrosine kinase inhibitors generally increase with the duration of treatment, but it still cannot be predicted.^[10]^

Patients with MV are prone to infection when their immunity is low, resulting in exudative pleural effusion. The inflammatory markers of infectious pleural effusion are elevated, and the pathogen may be found.

When tumor cells invade the pleura, patients with MV may develop a malignant pleural effusion, at which point tumor cells can be found in the pleural effusion. A quick and accurate diagnostic method that can be useful in the diagnosis of hematological disorders’ pleural localization is flow cytometry.^[11]^ Lymphoid pulp with characteristics of tumor cells and macroglobulin infringement of thoracic duct or pleural lymphatic vessel, MV patients can appear chylothorax.^[12]^

Patients with MV may also have transudative pleural effusion due to low serum albumin. The patients with pleural effusion caused by tyrosine kinase need to stop the drug, the patients with pleural effusion caused by tumor invasion should pay more attention to the treatment of the primary disease, and the patients with pleural effusion caused by infection need to find pathogens and active anti-infection treatment. Therefore, the differential diagnosis of pleural effusion in MV patients is essential. The patient had a definite diagnosis of MV, with bloody exudative pleural effusion. Flow cytometry suggested abnormal B-cell clones, and pleural biopsy suggested tumor cell infiltration, but no apparent pathogen was found. Therefore, the patient’s pleural effusion was considered to be caused by tumor cell infiltration, and a tyrosine kinase inhibitor was continued to treat the primary disease.

## 4. Conclusion

In conclusion, bilateral bloody pleural effusion is a rare complication of WM, and finding the etiology of pleural effusion is significant for the subsequent treatment and prognosis of patients.

## Acknowledgments

The authors appreciate the patient’s consent to present this case. This work was supported by the Natural Science Foundation of Ningbo (NO.2021J242), Zhejiang basic public welfare research projectand Key research (LGF21C010001) and development plan of Ningbo city (2023Z179).

## Author contributions

**Writing – original draft:** Tiantian Cen.

**Writing – review & editing:** Hongying Ma.

**Resources:** Qiaoli Zhang, Yanan Ying, Zhongbo Chen, Qian Deng, Gun Chen, Peipei Ye.

**Data curation:** Xiaoqun Zhang.

**Conceptualization:** Xiao Wu.

**Investigation:** Fengyun Tao.
